# Comparison of multi-parallel quantitative real-time PCRs targeting different DNA regions and detecting soil-transmitted helminths in stool

**DOI:** 10.1186/s13071-024-06464-6

**Published:** 2024-09-13

**Authors:** Marina Papaiakovou, Rubén O. Cimino, Nils Pilotte, Julia Dunn, D. Timothy J. Littlewood, Steven A. Williams, Alejandro J. Krolewiecki, Rojelio Mejia

**Affiliations:** 1 Department of Biological Sciences, Smith College, Northampton, MA 01063 USA; 2 Biodiversity & Health, Natural History Museum, Cromwell Road, London, SW7 5BD UK; 3 Department of Veterinary Medicine, University of Cambridge, Cambridge, CB3 0ES UK; 4 Instituto de Investigación de Enfermedades Tropicales (IIET), Universidad Nacional de Salta. Sede Regional Orán, Salta, Argentina; 5 Consejo Nacional de Investigaciones Científicas y Técnicas (CONICET), Buenos Aires, Argentina; 6 Department of Biology, Quinnipiac University, Hamden, CT 06518 USA; 7 Department of Infectious Disease and Epidemiology, Imperial College London, London, W2 1PG UK; 8 Section of Tropical Medicine, Department of Pediatrics, National School of Tropical Medicine, Texas Children’s Hospital and Baylor College of Medicine, Houston, TX 77030 USA

## Abstract

**Background:**

Soil-transmitted helminths infect an estimated 18% of the world’s population, causing a significant health burden. Microscopy has been the primary tool for diagnosing eggs from fecal samples, but its sensitivity drops in low-prevalence settings. Quantitative real-time polymerase chain reaction (qPCR) is slowly increasing in research and clinical settings. However, there is still no consensus on preferred qPCR targets.

**Methods:**

We aimed to compare soil-transmitted helminth (STH) DNA detection methods by testing naïve stool samples spiked with known quantities of STH eggs and larvae. DNA extracts from spiked samples were tested using independent quantitative realtime PCR (qPCR) assays targeting ribosomal or putative non-protein coding satellite sequences.

**Results:**

For *Trichuris trichiura,* there was a strong correlation between egg/larvae counts and qPCR results using either qPCR method (0.86 and 0.87, respectively). Strong correlations also existed for *A. lumbricoides* (0.60 and 0.63, respectively), but weaker correlations were found for *Ancylostoma duodenale* (0.41 for both assays) and *Strongyloides stercoralis* (0.48 and 0.65, respectively). No correlation for *Necator americanus* was observed when testing with either qPCR assay. Both assays had fair-to-moderate agreement across targets when using field-collected stool samples (0.28–0.45, for all STHs), except for *S. stercoralis* (0.12) with slight agreement.

**Conclusions:**

There is a strong correlation between qPCR results and egg/larvae counts. Our study confirms that qPCR is an effective diagnostic tool, even with low-intensity infections, regardless of the DNA-based diagnostic marker used. However, the moderate agreement between the two different qPCR assays when testing field samples highlights the need to understand the role of these targets in the genome so that the parasite burden can be quantified more accurately and consistently by qPCR.

**﻿Graphical abstract:**

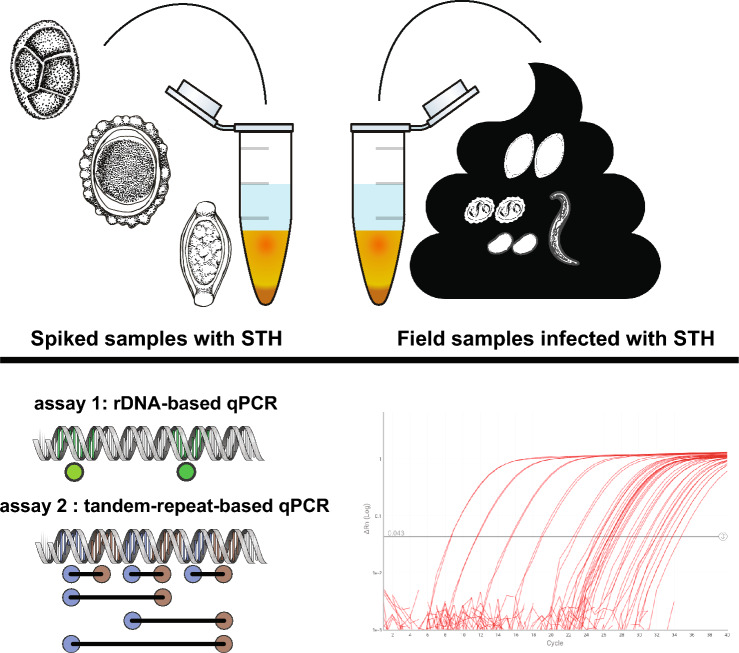

**Supplementary Information:**

The online version contains supplementary material available at 10.1186/s13071-024-06464-6.

## Background

Soil-transmitted helminths (STHs) (*Ascaris lumbricoides*, *Trichuris trichiura*, *Necator americanus*, *Ancylostoma duodenale*) and *Strongyloides stercoralis* infect more than 1.4 billion people worldwide [1], resulting in years of disability and extensive morbidity. The most widely used techniques for diagnosing STH infections are microscopy-based, despite the repeated demonstration of their shortcomings [2]. Microscopy is still recommended by the World Health Organization (WHO) for use in epidemiological interventions and monitoring progress in deworming programs [3]. Advances in molecular testing have spurred interest in developing new tools for monitoring STH infections, such as assays utilizing real-time polymerase chain reaction (qPCR) [4–6]. There is a need for more sensitive tools to complement WHO efforts to monitor the elimination of STHs [7]. Intervention success and impact assessment are highly reliant upon sensitive and accurate diagnostic tools for STH detection. However, attempts to evaluate molecular methods remain incomplete, including assessing one or more microscopic techniques against molecular methods [8–10]. Different real-time PCR assays targeting various DNA regions [ribosomal internal transcribed spacer sequences (ITS), ribosomal subunit sequences, or mitochondrial genes] have been developed to detect STHs [11]. Assays targeting mitochondrial and ribosomal sequences leverage their relatively high copy numbers, providing moderate-to-high sensitivity for real-time PCR. Ribosomal assays have been validated in numerous studies and clinical settings for STH detection [6, 8, 12, 13]. However, ribosomal targets tend to be conserved between species and present in lower copy numbers than other genome repeats. Given their conservation, they are frequently less specific than targets designed from other repeat types. Other nuclear tandemly arranged repeats can reach up to 37% of the parasite’s genome in certain species of STHs [14]. The development of new bioinformatics tools [15] and the assembly of improved genomes for the various STHs [16] has facilitated the exploration of the function of these target sequences and the genetic variation. Novel, optimized assays targeting highly repetitive elements in a parasite’s genome have the potential to reduce time and cost through high throughput automation [14]. Such targets have enhanced the sensitivity and specificity of qPCR assays, allowing for the differentiation of closely related species and facilitating target detection at copy numbers below those found within a single egg [5, 17].

This study assesses the agreement when two different molecular assays utilize different target sequences and compares results across a panel of samples spiked with known quantities of STH eggs or larvae. Similar testing was also performed on a panel of field-collected samples to assess the transferability of results.

## Methods

### Spiked sample preparation

Known numbers of parasitic eggs (1, 2, 5, 10, 15, 20, 40 egg or larvae) were used to spike 10 mg samples of naïve stool at Baylor College of Medicine (BCM), followed by DNA extraction using the FastDNA Spin Kit for Soil (MP Biomedicals, Santa Ana, CA) and a high-speed homogenizer (FastPrep-24, MP Biomedicals). In total, 19 samples containing *A. lumbricoides* eggs were created, as were 20 containing *T. trichiura* eggs, 24 containing *S. stercoralis* larvae, and 10 containing hookworm eggs. Details on the number of replicates per egg/larvae quantity used for spiking can be found in Supplementary Table S1. Aliquots of the same DNA extracts were shipped from the BCM to the Natural History Museum (NHM) for testing.

### qPCR testing

Two independent laboratories, BCM and NHM, tested aliquots of the same DNA extracts. The NHM assay was initially developed at Smith College in Northampton, MA, USA [5], and the BCM assay was initially developed at the National Institutes of Health (NIH) in Bethesda, MD, USA [6]. The assays used for testing target repetitive genomic elements, except for the *A. lumbricoides* assay, which targets the internal transcribed spacer 1 (ITS1) region. The assays used at BCM all target ribosomal genes (ITS1 for *A. lumbricoides*, ITS1 for *T. trichiura*, 18S for *S. stercoralis*, ITS2 for *N. americanus*, and ITS2 for *A. duodenale*).

### Field sample testing

A panel of 130 samples was collected as part of ongoing field studies in Orán, Argentina (approved by the bioethics committee of Colegio de Médicos de la Provincia de Salta and the IRBs of BCM; protocol number H-34926). All samples underwent direct smear stool microscopy and were frozen, without preservatives, until 50 mg of each sample was subjected to DNA extraction. Aliquots were sent to BCM and NHM for qPCR analysis using the above assays.

### Statistical analysis

The correlation between target concentration (fg/µl or copies/µl, for the BCM assays and the NHM assays, respectively) and spiked egg numbers was assessed by the Kendall rank correlation test [18]. Correlations were visualized in R v.4.2.2; *N. americanus* was excluded from the graphs due to too few data points, but the correlation values are still presented in Table 1. *P*-values < 0.05 were considered statistically significant. Comparisons between the two qPCRs were depicted using unweighted Cohen’s kappa agreement [19]. Fleiss kappa was calculated to evaluate the agreement between microscopy and the two qPCR tests (three raters), treating results as categorical values (presence/absence).

**Table 1:** Kendall correlation, as Tau-b value and respective *P*-values, between the number of larvae/eggs and the quantitative qPCR method for each soil-transmitted helminth for NHM and BCM assays

Species	Kendall Tau-b values: egg/larvae versus qPCR method			
	NHM	*P*-value	BCM	*P*-value
*Ancylostoma duodenale*	0.41	0.44	0.41	0.44
*Ascaris lumbricoides*	0.60	< 0.01	0.63	< 0.01
*Necator americanus*	0	1	−0.81	0.12
*Strongyloides stercoralis*	0.48	< 0.01	0.65	< 0.01
*Trichuris trichiura*	0.86	< 0.01	0.87	< 0.01

Kendall Tau-b values range between −1 (all pairs discordant) and 1 (all concordant); a higher Tau-b value indicates more concordance than discordant pairs of individual egg counts, and therefore, a higher overall correlation. Interpretation as <  + or −0.10: very weak; + or −0.10 to 0.19: weak; + or −0.20 to 0.29: moderate; and + or −0.30 or above: strong

## Results

### Concordance between qPCR and egg/larvae counts

The Kendall Tau-b values for the NHM and BCM assays were 0.86 and 0.87 for *T. trichiura* and 0.60 and 0.63 for *A. lumbricoides*, indicating strong concordance between DNA quantity measured using qPCR and egg numbers as determined by microscopy for both STHs. Using both assays, the Tau-b values for *A. duodenale* (0.41 for both, but not significant) and *S. stercoralis* (0.48 and 0.65, respectively) were less strong but still significant. With 0 and −0.816 Tau-b values for the NHM and BCM assays, respectively, results for *N. americanus* are probably due to ineffective extraction or insufficient eggs/larvae, making it difficult to draw any conclusions (Table 1). The graphs in Additional File 1: Fig. S1. also show the linearity and correlation between the qPCR quantitative method and eggs or larvae spiked (*N. americanus* graph not shown).

### Two- and three-rater agreement on field samples

We calculated the overall percentage agreement (total number of agreed positives or negatives in a given sample set). Cohen’s kappa for agreement on a sample-by-sample case, for both sets of qPCR assays, treats data as categorical values (presence/absence) since the qPCR output interpretation (i.e., quantitation) still does not correspond directly to worm burden or worm intensity [9, 20]. Fleiss kappa was calculated to show the greater discordance between microscopy and both qPCR assays; the results are presented in Table 2. Between the two sets of qPCR assays, there was a moderate agreement for *A. lumbricoides* (kappa = 0.43) and fair agreement for *N. americanus* (kappa = 0.33), *T. trichiura* (kappa value = 0.366), and *A. duodenale* (kappa value = 0.28). However, both assays showed a slight agreement for *S. stercoralis* (kappa value = 0.121). As expected, Fleiss kappa showed weak agreement between microscopy (ranging from 0.06 to 0.22) and qPCR assays (Table 2).

**Table 2:** Performance comparison between (i) NHM and BMC qPCR methods and (ii) between the two qPCRs and microscopy in field-collected stool samples

	Two qPCRs—BCM and NHM (two raters)			Fleiss kappa (three raters)	
	Unweighted Cohen’s kappa agreement	Percentage agreement	*P*-value	Fleiss kappa (categorical)	*P*-value
*Necator americanus*	0.33	82	< 0.01	0.21	< 0.01
*Trichuris trichiura*	0.37	96	< 0.01	0.18	< 0.01
*Ancylostoma duodenale*	0.28	64	< 0.001	0.15	< 0.01
*Strongyloides stercoralis*	0.12	77	< 0.001	0.06	0.02
*Ascaris lumbricoides*	0.45	92	< 0.001	0.22	< 0.01

Cohen’s and Fleiss kappas were calculated to evaluate the agreement between the two qPCR assays (two raters) and the agreement between microscopy and the two qPCR tests (three raters), respectively, treating results as categorical values (presence/absence). Kappa < 0 means no agreement; 0–0.20 slight agreement; 0.21–0.40 fair; 0.41–0.60 moderate; 0.61–0.80 substantial; and 0.81–1.0 perfect. All calculations for kappa (R package irr) and visuals for the correlations were conducted in R v.4.2.2

## Discussion

We present a comparative study evaluating two independent qPCR assay platforms for four STH species and *S. stercoralis* using laboratory-spiked and field-collected samples. We showed concordance and moderate-to-strong correlation between the presence of helminth eggs or larvae and the amount of parasite DNA. A strong correlation between spiked eggs and qPCR output has been demonstrated previously in similar settings [21]. Greater discordance between both qPCR platforms and microscopy illustrates the superior sensitivity (true positives) and specificity (true negatives) of the molecular methods compared with coprological tests. This comparative study demonstrates the benefits of qPCR when STH prevalence and intensity are low in a population. Molecular assays can be the foundation for reliable diagnostic results, irrespective of the target used, and opportunities for technological transfer, even to resource-limited areas, are expanding [22, 23]. We acknowledge that the number of spiked samples (eggs and larvae) used in this study was limited, requiring further scaling. This could partially explain the poor correlation observed for *A. duodenale*. The poor concordance between larval counts and qPCR for *S. stercoralis* could be explained by the low sensitivity of qPCR for *Strongyloides* [24]. We focused solely on spiking stool samples with small numbers of eggs/larvae, as the correlation between larger egg counts and qPCR in field-collected stool samples has already been demonstrated [7, 9, 25]. Another limitation was that an extraction control to check the efficacy of the DNA extraction method was not available at the time of this study. Suboptimal extraction of the samples spiked with *N. americanus* could explain the 0 and negative correlations for both *N. americanus-*targeting assays. Although DNA extraction products were sent to each institution, false negatives will impact both assays. One downside of using repeat-based assays is that the copy number of highly repetitive sequences can vary significantly between individual organisms and even within different stages of the same organism’s life cycle. However, larger monomer repeats might be more consistent within species [14, 26]. Further and future genomic work will highlight structural and geographical differences in repeat-based diagnostics. Currently, the full function of these nuclear repeats is mainly unknown. As a result, we are unsure how random mutations affect these areas and whether this variability can lead to bias in the quantification of STHs. These mutations make it challenging to compare results between samples accurately. Another limitation was the lack of egg/larvae counts from stool microscopy. However, previous studies have shown that the amount of egg burden directly correlates with detecting parasite DNA with qPCR in field-collected stool samples [6, 9].

## Conclusions

In summary, we present further evidence that qPCR is a valid alternative to fecal microscopy for the detection of STH, and our study supports current attempts [7] toward replacing coprological tools to assess low-intensity STH infections using qPCR. Results highlight assay-specific strengths and weaknesses. This study represents the first comparison of two distinct qPCR platforms with unique molecular targets. The study involved samples spiked with known quantities of STH eggs or larvae. The findings provide a framework for better understanding what constitutes a reliable diagnostic target. Developing standardized, accurate, and quality-controlled measures is crucial to successfully testing for STHs in fecal samples using qPCR. Standardization can be achieved through an External Quality Assessment Scheme (EQAS) program [27], which ensures consistency and diagnostic precision.

## Supplementary Information

Additional file 1.

## Data Availability

Data availability declaration in the manuscript.
