# Supplementary material for: Comparison of multi-parallel quantitative real-time PCRs targeting different DNA regions and detecting soil-transmitted helminths in stool
Source: Parasit Vectors. 2024 Sep 13;17:390. doi: 10.1186/s13071-024-06464-6 (PMC11397029; doi:10.1186/s13071-024-06464-6)
Supplement: Supplementary file 1 — Additional file 1. [file 13071_2024_6464_MOESM1_ESM.docx]

# Additional File 1

**
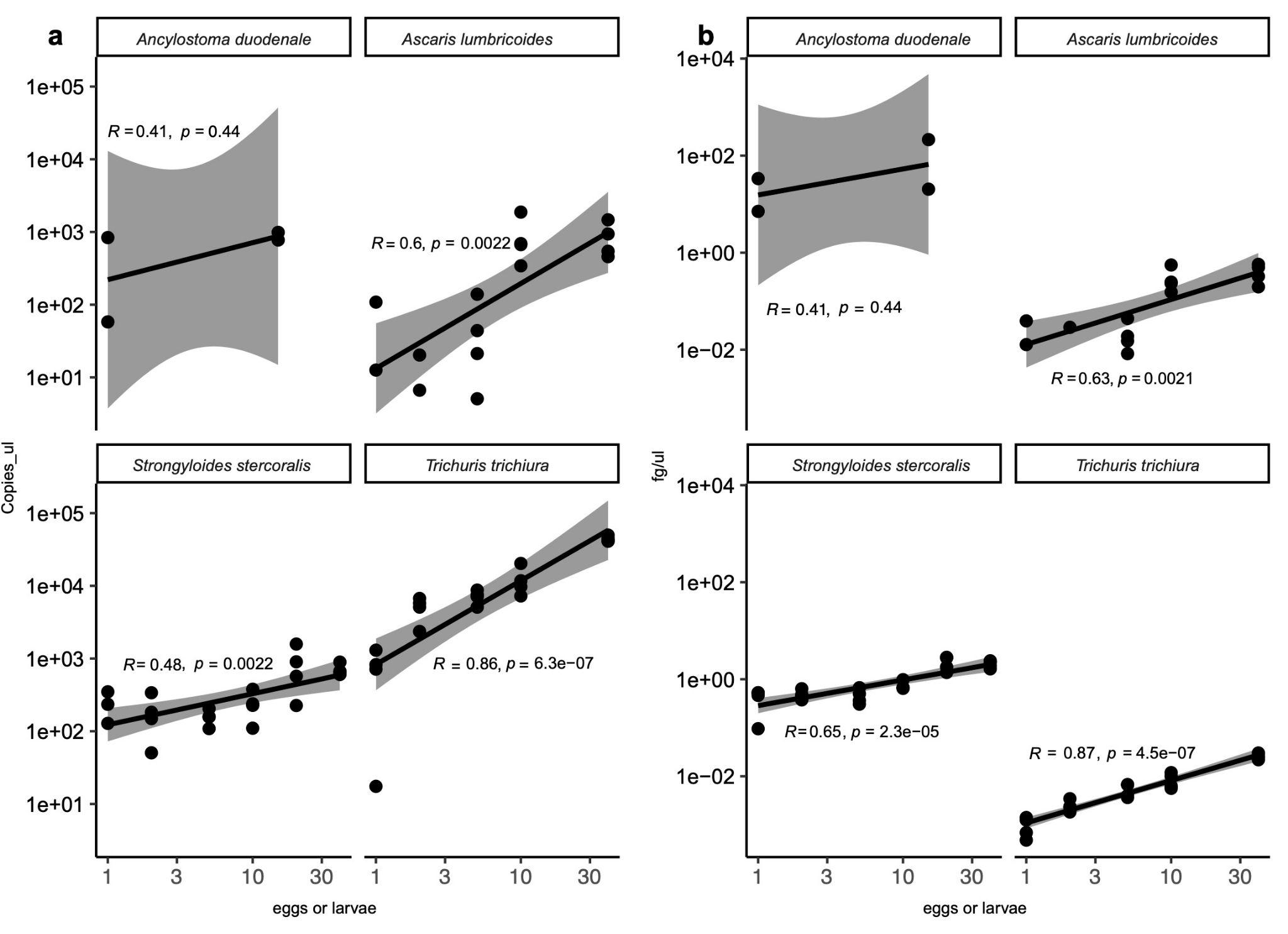
**

**Figure S1.** **Correlation between egg/larvae in spiked samples and qPCR method of quantitation**. **a**, Correlation between copies/µl and eggs/larvae-spiked samples for the NHM assays and **b,** correlation between fg/µl and eggs/larvae-spiked samples for the BCM assays. Correlations were calculated by the Kendall rank correlation test, with Kendall Tau-b values ranging between −1 (all pairs discordant) and 1 (all concordant); a higher Tau-b value indicates more concordance than discordant pairs of individual egg counts, and therefore, higher overall correlation. Interpretation as <  + or −0.10: very weak; + or −0.10 to 0.19: weak; + or −0.20 to 0.29: moderate; and + or −0.30 or above: strong
